# Mitogenomic analysis of a representative of the Chernyakhov culture in the Middle Dniester and their genetic relationship with the Slavs in the context of paleoanthropological data

**DOI:** 10.18699/vjgb-25-79

**Published:** 2025-09

**Authors:** E.V. Rozhdestvenskikh, T.V. Andreeva, A.B. Malyarchuk, I.Yu. Adrianova, D.S. Khodyreva, A.A. Evteev, A.P. Buzhilova, E.I. Rogaev

**Affiliations:** Research Center for Genetics and Life Sciences, Sirius University of Science and Technology, Sirius Federal Territory, Krasnodar region, Russia; Research Center for Genetics and Life Sciences, Sirius University of Science and Technology, Sirius Federal Territory, Krasnodar region, Russia Centre of Genetics and Genetic Technologies, Faculty of Biology, Lomonosov Moscow State University, Moscow, Russia Vavilov Institute of General Genetics of the Russian Academy of Sciences, Moscow, Russia; Centre of Genetics and Genetic Technologies, Faculty of Biology, Lomonosov Moscow State University, Moscow, Russia Vavilov Institute of General Genetics of the Russian Academy of Sciences, Moscow, Russia; Vavilov Institute of General Genetics of the Russian Academy of Sciences, Moscow, Russia; Research Institute and Museum of Anthropology, Lomonosov Moscow State University, Moscow, Russia; Research Institute and Museum of Anthropology, Lomonosov Moscow State University, Moscow, Russia; Research Institute and Museum of Anthropology, Lomonosov Moscow State University, Moscow, Russia; Research Center for Genetics and Life Sciences, Sirius University of Science and Technology, Sirius Federal Territory, Krasnodar region, Russia Department of Psychiatry, UMass Chan Medical School, Shrewsbury, MA, USA

**Keywords:** Chernyakhov culture, Slavs, ancient DNA, mitochondrial DNA, H5a1a1, craniology, phylogeographic analysis, черняховская культура, славяне, древняя ДНК, митохондриальная ДНК, H5a1a1, краниология, филогеографический анализ

## Abstract

Occupying a fairly extensive territory within the East European Plain, representatives of the Chernyakhov culture interacted with many synchronous tribes of other cultures inhabiting neighbouring regions. The question of a possible Proto-Slavic component in the population of the Chernyakhov culture is a subject of many years of discussion, but there is still no evidence for the genetic contribution of representatives of this culture to the gene pool of the Slavs in the subsequent historical period. In this study, we present the results of the craniological and genetic analysis of an individual from the Krynichki burial ground, presumably belonging to the Slavic part of the population of the Chernyakhov culture. A craniometric comparative analysis was conducted for several series of skulls of the East Slavs and representatives of the Chernyakhov culture. The comparison of intragroup variability in the groups of the two cultures showed marked differences between them in the first three principal components. At the same time, the East Slavic and Chernyakhov cultures have similar levels of craniological variability. Differences between female specimens are not so pronounced as those of males’. Based on the analysis of whole-genome sequencing data, the individual from the Krynichki was identified as being a female. The complete sequence of mitochondrial DNA, which belongs to the haplogroup H5a1a1, was reconstructed. For this mitochondrial lineage, a phylogenetic relationship was revealed with eight specimens from publicly available genomic databases, five of which belong to representatives of the present-day West and East Slavic populations. Furthermore, we revealed a mitochondrial sequence identical to that from our previous research on an individual from a medieval burial site located in the modern Vologda region, which is thought to have Slavic ancestry. The complete match between the medieval individual’s mtDNA sequence and that of a representative of the Chernyakhov culture points to their likely maternal ancestry. Thus, a possible continuity between representatives of the Chernyakhov culture (3rd century AD) and the population of Ancient Rus’ (the second half of the 12th–early 13th centuries AD) has for the first time been shown, as genomic data suggest.

## Abstract (ru)

Занимая довольно обширную территорию в пределах Русской равнины, представители черняховской культуры взаимодействовали со многими синхронными племенами, принадлежащими к другим культурным группам и населявшими смежные регионы. Предметом многолетних дискуссий является вопрос о возможном присутствии протославянского компонента в структуре населения черняховской культуры, однако до сих пор отсутствует доказательная база генетического вклада представителей данной культуры в генофонд славян в последующем историческом периоде. В статье представлены результаты краниологического и генетического анализа индивида из могильника Кринички, предположительно, относящегося к славянской части населения черняховской культуры. Проведен краниометрический сравнительный анализ нескольких серий черепов восточных славян и представителей черняховской культуры. Сопоставление внутригрупповой изменчивости в группах двух культур показало заметные различия между ними по трем первым главным компонентам. В то же время диапазон краниологической изменчивости восточных славян и носителей черняховской культуры сопоставим. Различия в женских выборках не так резко выражены, как в мужских. На основе анализа данных полногеномного секвенирования определен женский пол исследуемого индивида. Реконструирована полная последовательность митохондриальной ДНК, которая относится к гаплогруппе H5a1a1. Для этой митохондриальной линии выявлена филогенетическая связь с восемью образцами, найденными в открытых базах данных геномных последовательностей, пять из которых относятся к представителям современных западно- и восточнославянских популяций. Более того, обнаружена идентичная митохондриальная последовательность, принадлежащая исследованному нами ранее индивиду из средневекового могильника на территории современной Вологодской области, для которого рассматривается славянское происхождение. Полное совпадение последовательности мтДНК представителя черняховской культуры и этого средневекового индивида предполагает их вероятное родство по материнской линии. Таким образом, впервые на основе геномных данных показана возможная преемственность между некоторыми представителями черняховской культуры (III в. н. э.) и населения Древней Руси (вторая половина XII–начало XIII вв.).

## Introduction

A new cultural entity, known as the Chernyakhov culture, arose at the end of the Roman era in the territories of the Northern Black Sea Region and the upper reaches of the Dniester-Western Bug rivers (Magomedov, 2001). The multi-ethnic origin of this group is currently the prevailing theory among experts (Sedov, 1979; Magomedov, 2001; Zinkovskaya, Kolesnikova, 2020). Nonetheless, it remains ambiguous which “barbarian” tribes formed the basis of the Chernyakhov culture, and which ones exerted influence subsequent to its final formation (Shchukin, 2005). Archaeologists examine the existence of a Slavic or Proto-Slavic element in its composition, the carriers of which thereafter developed the areas previously occupied by the representatives of the Chernyakhov culture (Sedov, 1979; Shchukin, 1997; Terpilovsky, 2000). The presence of Early Slavic cultural components can be seen in the Chernyakhov settlements in the Middle Dniester region (Lyapushkin, 1968; Rickman, 1975; Vinokur, 2002); however, pinpointing the purported Proto-Slavic component is a challenging objective when relying solely on archaeological and anthropological approaches (Terpilovsky, 2000; Magomedov, 2001).

Krynichki is a burial site located in the Middle Dniester, specifically in the Balti district of the Odessa region. As an archaeological site, it has been known since the end of the 19th century, but it began to be considered as a site of the Chernyakhov culture in the 20th century (Gamchenko, 1911; Symonovich, 1960). The existence of the Chernyakhov culture in the 3rd-4th centuries AD was verified by the artefacts found during the 1957–1958 excavations at this location carried out by the South Russian Expedition of the IIMK of the USSR Academy of Sciences. During the works on the Labushna Posad gully, a single-layer settlement and burial ground dated to the 3rd-4th centuries AD were discovered (Symonovich, 1960). There were only indications of inhumation in the burial. This characteristic distinctly differentiates this burial from the sites of the Chernyakhov culture, which are characterised by varying proportions of both inhumation and cremation rites (Nikitina, 1985). Furthermore, the grave was positioned separately from the others, which cannot be explained by family ties or belonging to a particular social group (Symonovich, 1960). In the burial site, only three of the five found skeleton remains burial site were quite well preserved, allowing for their anthropological and genetic investigations.A young girl individual from Burial 4 was studied in this research. The skeleton was found in an oval-shaped grave, lying on its back in an extended position, with the head orientated northeast. The burial contained two garter-style bronze crossbow fibulae, glass beads, a multi-part bone comb secured with bronze nails, and a cylindrical clay spindle adorned with a circular pattern on its sides. Also in the burial at the left elbow was a bronze staff-like pin, which apparently served to attach the ribbon to the braid (Nikitina, 2008). The aforementioned inventory is typical of female graves. It is significant that the habit of embellishing the braid with a ribbon was prevalent among East Slavic women up to the 19th-20th centuries (Chistov, 1987). This trait may be thought as indirect indication of the buried female’s relationship to the Slavic community

Complex genetic research of the Chernyakhov culture representatives, together with anthropological data, can aid in estimating their potential genetic contribution to the formation of Slavic populations. Previously, mitochondrial genome analysis has proved its effectiveness to obtain information about historical processes, particularly migratory occurrences (Andreeva et al., 2024), as well as to assess the kinship between individuals (Andreeva et al., 2023a) and the probable origins of the studied people (Andreeva et al., 2023b).

The purpose of this study was to conduct a comprehensive analysis of the presence of the Slavic component in Chernyakhov culture representatives using paleoanthropological and genetic data.

## Materials and methods

The skulls of 153 Chernyakhov culture representatives from the funds of the Research Institute and Museum of Anthropology of Lomonosov Moscow State University were measured and analysed; for comparative analysis by statistics methods, several craniological series of East Slavs (229 skulls) were also studied (Table 1). A part of the studied materials included in the analysis overlapped geographically. This is because the Slavic tribes inhabited far more territory than the Chernyakhov groups

**Table 1.**
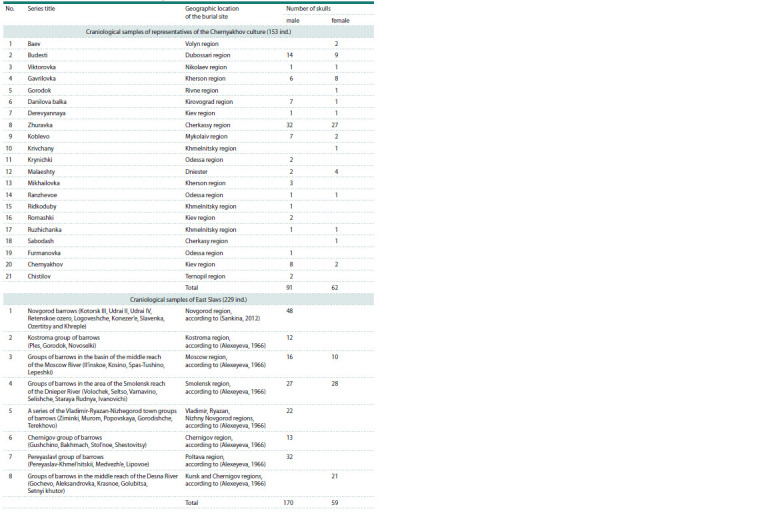
Craniological series used in anthropological analysis

Eight measurements of the facial section of the skull (Alekseev, Debets, 1964) were used in the analysis: mean facial width (46 Mar.), upper facial height (48 Mar.), orbital width (51 Mar.), orbital height (52 Mar.), nasal width (54 Mar.), subspinale height above the zygomaxillary chord, simotic width (SC Biom.) and height (SS Biom.). By World PCA software and the set of additional analytical techniques based on it, craniometric data were statistically analysed using the principal component (PC) approach (for a thorough description, see (Evteev et al., 2021)).

**Fig. 1.**
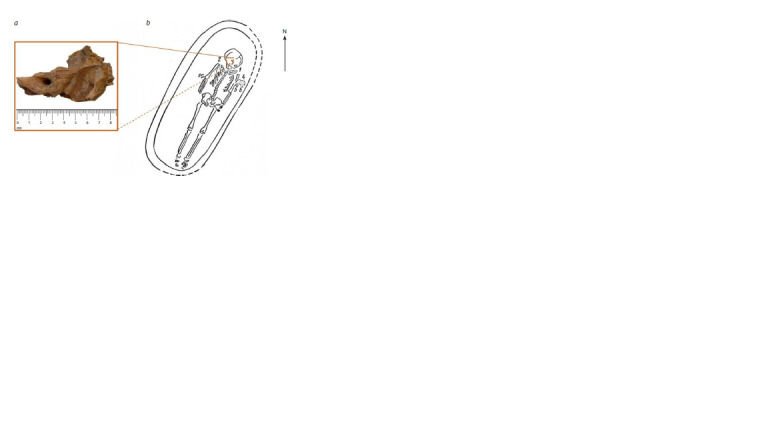
Anthropological material used for genetic analysis. a – the petrous part of right temporal bone of female individual (museum No. 10917); b – scheme of burial 4 from the burial site in the settlement of Krynichki (Symonovich, 1960).

A fragment of the petrous part of temporal bone of museum specimen No. 10917 (Fig. 1a) was used for genetic research. It belongs to a juvenile individual from grave 4 of the archaeological site of Krynichki (Fig. 1b). This burial site dates back to 230–270 AD (Nikitina, 2008).

Ancient DNA was extracted from the cochlea specimen weighing 0.236 grams. The DNA extraction was carried out in sterile rooms of the Sirius University of Science and Technology in accordance with the previously published methodology (Andreeva et al., 2022). The sample of DNA was assigned the number AB93.

The DNA quality was evaluated using an Agilent Bioanalyzer 2100 using a High Sensitivity DNA chip kit (“Agilent”). The ancient DNA was used to create a genomic library (Gansauge et al., 2017), which was then sequenced on the Illumina HiSeq 2500 platform using single-end reads.The bioinformatics analysis of the sequencing data involved multiple stages. AdapterRemoval v2 (Schubert et al., 2016) was used to remove adapter sequences. The nucleotide sequences with a length greater than 30 nucleotides were aligned to the reference genomes rCRS/NC_012920.1 (mitochondrial genome) (Anderson et al., 1981) and hg19/GRCh37 (human reference genome) using the BWA tool (Li, Durbin, 2009). MapDamage v.2.2.1 (Jónsson et al., 2013) was used to validate the authenticity of ancient DNA by analysing the frequency of C→T substitutions at the ends of reads. Contamination was evaluated using the Schmutzi software (Renaud et al., 2015). To identify the genetic sex of the sample, the ratios of the average number of reads covering sex chromosomes (X and Y separately) to the number of reads covering autosomal ones were calculated.Reads with a mapping quality score greater than 20 were used to reconstruct the mitochondrial sequence (mtDNA). The genetic variations were determined by the BCFtools program (Danecek et al., 2021). Additionally, quality filtering was applied to the genotypes (QUAL > 30), as well as normalisation of the identified insertions and deletions. The nucleotide variations found in mitochondrial DNA were verified using the IGV (integrative genomics viewer) tool (Robinson et al., 2011).Mitochondrial haplogroup determination of AB93 was performed using Haplogrep3 (Schönherr et al., 2023), based on Phylotree build 17 (Van Oven, 2015). The Yfull MTree 1.02 database (https://www.yfull.com/mtree/) was used for validation.

The search for the mtDNA sequences of both present-day and ancient individuals that had the highest similarity to the AB93 sample was conducted using public databases such as NCBI (https://www.ncbi.nlm.nih.gov/nuccore), Allen Ancient DNA Resource (AADR) (Mallick et al., 2024), Yfull MTree 1.02 (https://www.yfull.com/mtree/), and AmtDB (Ehler et al., 2019). The BLAST service (https://blast.ncbi. nlm.nih.gov/Blast.cgi) was used to search and select sequences in the NCBI database. The search conditions were set to 100 % Query coverage and a Percent Identity of at least 99.98 %.

The phylogeographic analysis was carried out using the maximum likelihood method in the mtPhyl program (Eltsov, Volodko, 2016). The major clade was constructed by grouping samples with the fewest number of nucleotide substitutions, taking into consideration the whole mitochondrial sequence. For samples from the ancestral haplogroup poly-C tracts, tandem repeat sections 522–524 and 573–576, and nucleotide position 16519 (a so-called “hot spot”) were excluded from the analysis.

## Results

**Fig. 2.**
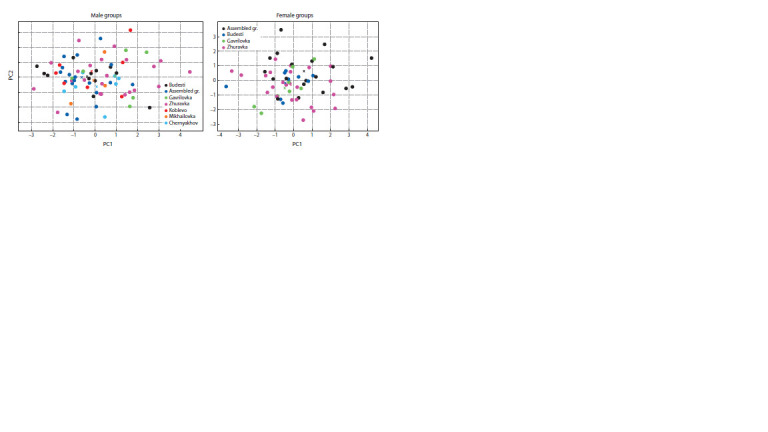
Location of the studied groups of the Chernyakhov culture in the space of PC1 and PC2.

At the first stage, several craniological series of East Slavic and Chernyakhov culture skulls were examined. On the basis of Chernyakhov male skulls, it was found that the first two principal components (PC1 and PC2) account for 47.5 % of the variability, while the first four ones account for 78.5 %. Therefore, the first two components show notable variance (Fig. 2). Individuals with high PC1 values have increased face height and width and facial profile and, to a lesser extent, increased eye socket height and nose width. Individuals with high PC2 values are characterised by a decrease in facial width combined with an increase in the size of the back of the nose. In general, the analysed groupings do not form clearly defined clusters. Nevertheless, the calculation of intragroup average pairwise Euclidean distances by PC1–4 values (SPER) showed that the samples from Budesti and Chernyakhov (left bank of the Dniester River, present-day Dubossari district, and present-day Kiev region, Obukhov district, respectively) are the most homogeneous (SPER ≤ 2.7), while the series from Gavrilovka (present-day Kherson region) is significantly more heterogeneous (SPER ≤ 4.5).

In the study of female skulls, the first two principal components explain 46 % of the overall variability, whereas the first four explain 73.7 %. The morphological meaning of PC1 is completely consistent with that of the male skulls: individuals with high values of this component have increased facial size, eye socket and nose width, as well as enhanced horizontal profiling. PC2 has a similar connotation in general, describing a rise in the size of the nasal bones. Thus, the basic tendencies of morphological diversity of the facial region of the skull in the male and female sections of the combined series of the Chernyakhov culture are similar (Fig. 2).

**Fig. 3.**
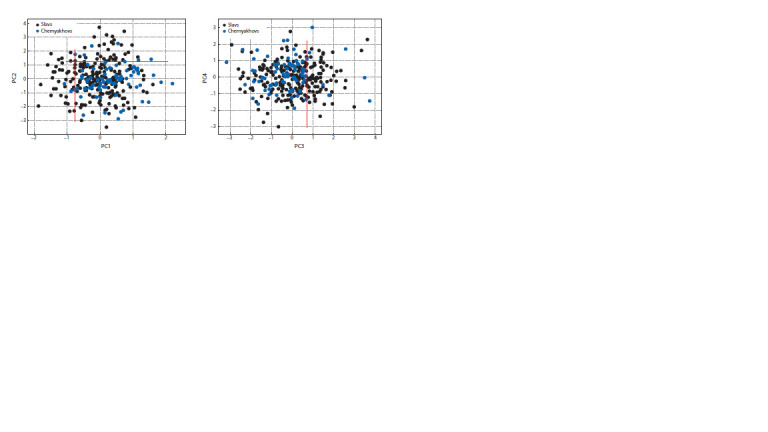
Location of the studied male skulls in the space of four PCs (East Slavs and representatives of the Chernyakhov culture together). The red lines in the figure restrict the cluster of East Slavs and Chernyakhov culture individuals with similar PC scores from the cluster of East Slavs with very different scores.

Additionally, principal component analysis was applied to the representatives of the East Slavs and the Chernyakhov culture. This analysis revealed substantial discrepancies between them for the first three PCs (Fig. 3). According to the Kaiser criterion (Deryabin, 2008), the first three principal components can be considered significant, as well as conditionally the fourth (eigenvalue 0.97). At the same time, East Slavs and individuals of the Chernyakhov culture have a similar range of overall craniological diversity (intragroup SPER – 3.18 and 3.07, respectively). Some local male series of skulls (Budeshti and Chernyakhov) overlap with East Slavic groupings (Smolensk, Kostroma, and Vladimir-Ryazan-Nizhegorod regions). The disparities in the generalised female series of skulls are less pronounced than those observed in the male series.

The second stage was genetic analysis of AB93. The whole mitochondrial DNA sequence of the Chernyakhov culture representative was reconstructed. This individual presumably has a Proto-Slavic component based on the archaeological context.

**Fig. 4.**
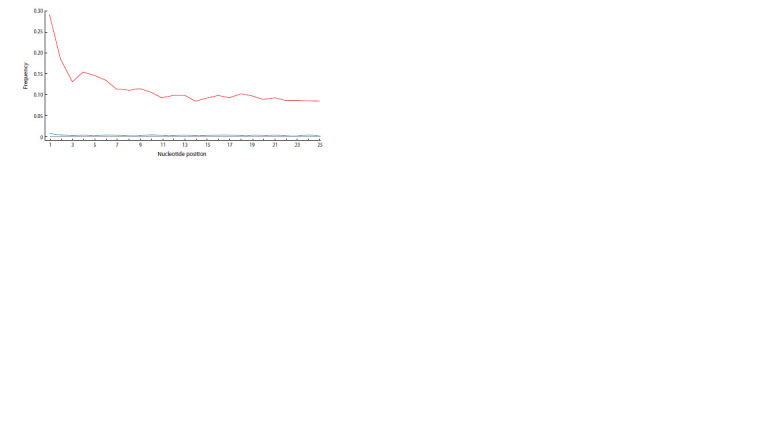
Nucleotide substitution profile obtained using the mapDamage2 programme (Jónsson et al., 2013) for reads mapped to the mitochondrial reference genome. The red line indicates specific for ancient DNA C→T substitutions among the first 25 nucleotides from the 5’-end of the DNA fragments.

According to the results of sequencing and primary bioinformatic analysis, about 115.8 million short reads were obtained, of which 51.5 % were mapped to the human reference genome. The higher frequency of C→T substitutions observed throughout the entire length of the fragments (Fig. 4) confirms that the AB93 sample belongs to ancient DNA. This specific DNA deamination deals with postmortem alterations and marks DNA extracted from archaeological and anthropological samples well. The ratio of the average number of reads covering sex chromosomes (X and Y separately) to the number of reads covering autosomal ones revealed that the studied sample belonged to a female, which is consistent with the archaeological context.

Mapping reads to the reference human mitochondrial genome (NC_012920.1) resulted in the reconstruction of the whole mitochondrial DNA sequence. The average coverage was x48,32, which allowed us to identify the mitochondrial haplogroup of AB93 and to conduct phylogeographic analysis. The analysis of the AB93 mtDNA sequence revealed variations associated with haplogroup H5a1a1 (Table 2).

**Table 2.**
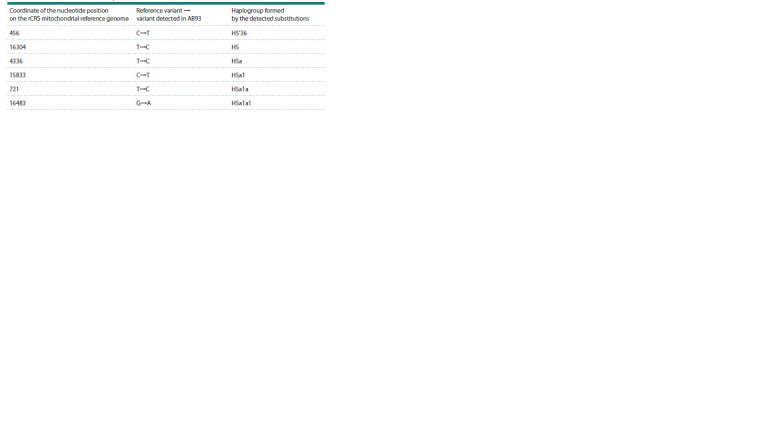
Variants of single nucleotide substitutions, forming mitochondrial haplogroups and detected in the mitochondrial sequence of AB93

In addition to haplogroup-forming variations, the mtDNA sequence of AB93 had an A-to-G substitution at position 93 located in hypervariable region 2 (HVR2). This variant does not define a haplogroup and is a private variant of the AB93 sample.

Mitochondrial sequences of individuals from public databases belonging to haplogroups H5a1a and H5a1a1 were used for phylogeographic analysis. Additionally, mtDNA sequences diverging by no more than three haplogroup-forming substitutions were included in the analysis. Thus, the sequences of 38 samples, including AB93, with geographic, ethnic, or cultural affiliation were selected for phylogeographic analysis. Of these, three sequences belonged to ancient individuals (Table 3) and 35 were from present-day individuals

**Table 3.**
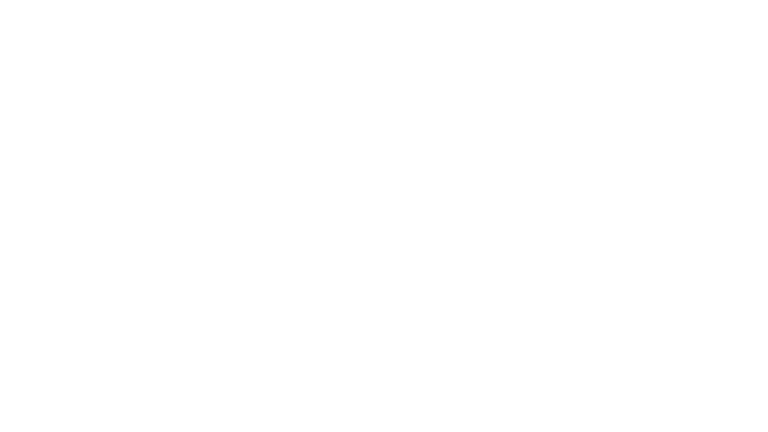
Ancient samples with mitochondrial DNA belonging to haplogroup H5a1a * Haplogroup is indicated according to the PhyloTree consensus classification; samples not included in the phylogeographic analysis are marked in grey.

**Fig. 5.**
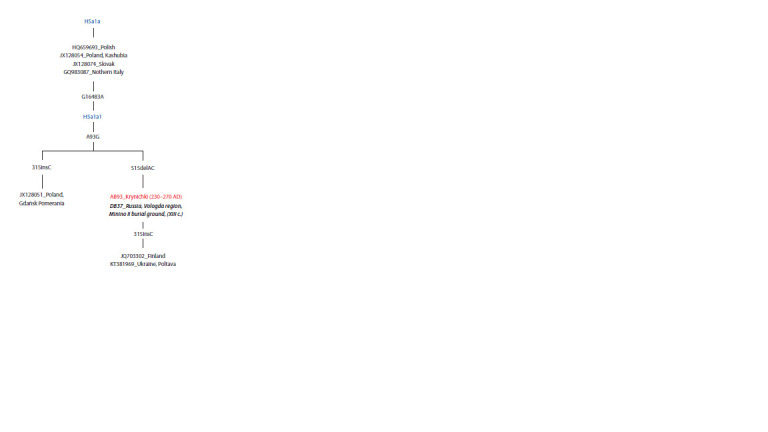
Fragment of the phylogeographic tree constructed using the mtPhyl program for mtDNA samples of the mitochondrial branch of H5a1a. The studied sample is shown in red. The ancient individual highlighted in bold italics. Present-day samples have an identifying number as well as geographic/ ethnic information. Haplogroups of the H5a1 lineage are indicated in blue. For transitions, the position number and the substitution variant are indicated. ins – insertion and its location in the genome; del – deletion and its location in the genome.

Figure 5 shows a fragment of the reconstructed phylogeographic tree of the mitochondrial lineage H5a1a. Five out of the eight samples belong to representatives of present-day Slavic populations and one is a previously studied individual from a medieval burial site in the modern Vologda region. This individual was dated to the latter half of the 12th to early 13th centuries AD and was identified as having Slavic origins (Rozhdestvenskikh et al., 2024). The mitochondrial DNA sequences of a Chernyakhov culture representative (AB93) and a medieval individual (DB37) from the Minino II burial site were found to be identical

## Discussion

The results of the anthropological analysis demonstrate that the morphological variability of the Chernyakhov culture’s individuals has a generally uniform character in the space of principal components, with no pronounced clusters. At the same time, the overall range of craniological variability is significant and comparable to that in generalised craniological series for the entire territory of the settlement of the medieval East Slavs. This result indicates that the craniological series of Chernyakhov representatives have heterogeneity, potentially attributable to genetic factors

The mitochondrial sequence of the studied individual of the Chernyakhov culture belongs to haplogroup H5a1a1. This lineage is part of the ancestral clade H5a1a, which is prevalent in present-day Slavic groups in Central and Eastern Europe (Mielnik-Sikorska et al., 2013; Malyarchuk et al., 2017). It was found in East Slavic populations (including Russians, Ukrainians, and Belarusians) as well as among West Slavs (Poles, Czechs, Slovaks, and Kashubians) (Mielnik-Sikorska et al., 2013; Malyarchuk et al., 2017). Haplogroup H5a1a has the highest frequency (13.29 % of the entire sample) among present-day Poles (https://www.familytreedna.com/).All ancient individuals whose mitochondrial sequences belong to haplogroup H5a1a have been found in the territory of Eastern Europe. The oldest bearer of this haplogroup dating back to the Early Bronze Age was found in present-day Bulgaria, in the eastern part of the Balkan Peninsula (Thrace) (Modi et al., 2019). In Poland, haplogroup H5a1a was found in a representative of the Wielbarka culture (100–300 AD) (Stolarek et al., 2023), as well as in a medieval individual from the Santok necropolis in western Poland. This necropolis is assigned to the local Pomeranian people and dates back to the 11th–12th centuries AD (Stolarek et al., 2023). The hypothesis has been proposed that the Wielbark civilisation was based on the indigenous population of the Vistula and Western Bug river basins, as well as Gothic tribes who migrated from southern Scandinavia (Stolarek et al., 2023). Furthermore, the local tribes are thought to represent Eastern Europe’s Proto-Slavic population (Grzesik, 2017). Significantly, an analysis of the hypervariable regions of mitochondrial sequences demonstrated the continuity of mitochondrial lineages in present-day Poland, dating back at least to the Roman period. It was also found that the local maternal lineages are part of the mitochondrial branch H5a1 (Juras et al., 2014).

We revealed that the mitochondrial DNA sequence of the representative of the Chernyakhov culture from the Middle Dniester exactly matches that of a medieval young man from the Russian North (present-day Vologda region) (Rozhdestvenskikh et al., 2024). It is important to note that, despite the fact that this individual from the Russian North was buried in a region primarily inhabited by local Finno-Ugric tribes, his burial customs followed Christian practices (Archaeology…, 2007). Also, this period is characterised by active interaction between Slavic and Finno-Ugric groups in the early stages of the Ancient Rus’ state formation. Most likely, this young man was a non-local Slavic representative (Rozhdestvenskikh et al., 2024). The identity of the mitochondrial sequences suggests a probable maternal relationship between these two individuals – a teenage girl from a burial in the south of Rus’ and a young man from a northern burial ground. This allows us to identify potential migration routes of the ancient population within the East European Plain

## Conclusion

As a result, our craniological analysis shows that East Slavs and Chernyakhov culture representatives share a similar range of craniological diversity. Genetic analysis also reveals that the mitochondrial lineage identified in the individual of the Chernyakhov culture is characteristic of Slavic groups, both present-day and ancient, who inhabited territories associated with the probable origin and settlement of the Slavs. In this regard, we can assume the presence of a genetic connection of the maternal lineage between representatives of the Chernyakhov culture and the ancient population of Eastern Europe, on the basis of which the Slavic community was formed. However, it should be noted that the data on the genetic connection between representatives of the Chernyakhov culture and the Slavs that we obtained for the first time on the basis of mitochondrial genomes require further confirmation using additional genetic markers and anthropological material.

## Conflict of interest

The authors declare no conflict of interest.
